# Supplementary material for: Determining factors of neonatal mortality in Ethiopia: An investigation from the 2019 Ethiopia Mini Demographic and Health Survey
Source: PLoS One. 2022 Dec 30;17(12):e0267999. doi: 10.1371/journal.pone.0267999 (PMC9803101; doi:10.1371/journal.pone.0267999)
Supplement: Attachment — Submitted filename: Rebuttal letter.docx [file pone.0267999.s001.docx]

Dear, Sidrah Nausheen, FCPS

Academic Editor

PLOS ONE

November 7, 2022

Thank you for allowing us to submit the revised manuscript [PONE-D-22-11460] to the PLOS ONE journal. We appreciate you and the reviewers for giving us your precious time in reviewing our paper and providing valuable and constructive comments. We have been carefully incorporated the comments and suggestions provided by the reviewers and academic editor, and tried our bests to address all of them. We hope the manuscript after careful revisions meet PLOS ONE high standards and requirements.

Below is point-by-point responses to reviewer comments and we have highlighted yellow to the changes within the manuscript.

\#**Reviewer1**:

Thank you so much for your detailed review of our manuscript, and for your constructive comments to our manuscript. We have tried our bests to incorporate them as follows.

1. The information related to study design of EMDHS 2019 was written under Sampling Techniques section.
2. We have included information about the software we have used for analysis
3. The table was restricted to Odd Ratio, its confidence interval and p-values only
4. We have removed the empety brackets.

**#Reviewer2:**

Thank you so much for your detailed review of our manuscript, and for your suggestions about the valuablity of the manuscript for policymakers. By saying this, we have tried our bests to check the spelling and grammar.
